# Effects of Metformin on Risk and Prognosis of Biliary Tract Cancer: A Systematic Review and Meta-Analysis

**DOI:** 10.3390/medicina59020298

**Published:** 2023-02-06

**Authors:** Junhong Chen, Hengwei Jin, Hao Zhou, Kai Liu

**Affiliations:** Department of Hepatobiliary and Pancreatic Surgery II, General Surgery Center, The First Hospital of Jilin University, Changchun 130021, China

**Keywords:** BTC, metformin, risk, prognosis, meta-analysis

## Abstract

*Background and Objectives*: Metformin has been found to potentially reduce the risk and improve the prognosis of a variety of tumors, but these findings remain controversial in biliary tract cancer (BTC). Therefore, this systematic review and meta-analysis was conducted to investigate the association between metformin and BTC. *Materials and Methods*: Two independent researchers comprehensively searched PubMed, Embase, the Cochrane Library, and Web of Science for eligible studies published from their inception to 31 March 2022. Comparisons of risk, overall survival (OS), and disease-free survival (DFS) for patients with BTC were selected as the endpoints of interest and pooled by random or fixed-effects models. *Results*: Eleven studies with a total of 24,788,738 participants were eligible for this analysis. The overall pooled effects showed no significant differences in biliary tract cancer risk (hazard ratio (HR) = 0.82, 95% confidence interval (CI): 0.50–1.35, *p* = 0.436), OS (HR = 0.88, 95% CI: 0.74–1.04, *p* = 0.135), or DFS (HR = 1.03, 95% CI: 0.79–1.34, *p* = 0.829) between metformin users and non-users. When restricting participants to those with diabetes, a similar negative result was found, demonstrating that metformin use was not significantly associated with a lower risk of developing BTC compared with a lack of metformin use (HR = 0.65, 95% CI: 0.39–1.07, *p* = 0.089); notably, the included studies exhibited significant heterogeneity in the selection of participants and the definition of metformin users. *Conclusions*: Metformin may not be able to reduce the risk of BTC and improve prognosis in certain populations. Based on the limited quantity and quality of the included studies, the present results should be interpreted within their limitations, and further studies are warranted to determine the optimal timing, dose, duration, and scenario of metformin administration.

## 1. Introduction

Biliary tract cancer (BTC), classified as gallbladder cancer, intrahepatic cholangiocarcinoma (ICC), and extrahepatic cholangiocarcinoma, originates from the epithelial cells of the biliary tree and involves the biliary system [1,2]. It used to be considered a rare tumor; however, its incidence has continuously risen in recent decades, and it is now the second most common malignancy of the hepatobiliary system after hepatocellular carcinoma [3]. The annual incidence of ICC is 0.85 per 100,000, and it is the second most common type of primary liver cancer [4]. The five-year survival rate for all subtypes of BTC is below 10%, since BTC is highly malignant and always detected at an advanced stage [2,5]. While specific clinical symptoms are uncommon in the early stages, jaundice, abdominal pain, and weight loss may develop in the advanced stages. Due to its insidious onset and rapid progression, the cancer has often progressed to an advanced stage at the time of diagnosis [1]. Its treatment is challenging, with surgery as the primary treatment; available systemic therapies, including gemcitabine and cisplatin, have limited efficacy and are treatment options for advanced diseases [6,7]. In recent years, a deeper understanding of the biological molecular landscape of BTC is rapidly driving the development of a variety of new molecular target agents, such as anti-human epidermal growth factor receptor (HER)-2 antibodies, isocitrate dehydrogenase (IDH)-1 inhibitors, and fibroblast growth factor receptor (FGFR)-2 inhibitors; immunotherapies represented by immune checkpoint inhibitors (ICIs) are also rapidly changing the treatment scenario for BTC [8,9]. However, the efficacy of these emerging therapies in unselected patients with BTC remains limited [8,9]. Therefore, preventing BTC and developing novel therapeutic options are essential in addressing this highly malignant disease that is becoming a serious public health problem for families, the healthcare system, and society as a whole [3].

Diabetes is a risk and a poor prognostic factor for several tumors, and previous studies have identified its adverse effects on BTC [10,11,12]. Compared to non-diabetic individuals, patients with type 2 diabetes have an increased risk and worse prognosis for BTC [10,11,12]. Recent studies have also found that the prevalence and co-occurrence of diabetes is higher in digestive cancers [13,14]. Metformin is often used as the first-line treatment for type 2 diabetes [15]. It not only controls patients’ blood glucose through multiple pathways, but also has anti-inflammatory and insulin-lowering effects [16]. In recent years, the potential of non-anticancer drugs in cancer prevention and treatment has attracted increasing interest from researchers; there are substantial data demonstrating the role of metformin in reducing the risk of various tumors and improving patient prognosis [17]. Several pre-clinical studies on BTC have also established that metformin may exert its antitumor activity by inhibiting protein synthesis, blocking the cell cycle and tumor angiogenesis, and sensitizing cancer cells to chemotherapeutic agents [18,19,20]. However, published clinical and population-based studies have had contradictory results, and the chemotherapeutic and preventive potential of metformin in BTC remains controversial. For example, previous studies have shown that patients with type 2 diabetes treated with metformin appear to have a decreased risk of BTC [21,22], while some evidence has found no association between metformin usage and BTC [23,24]. There is no consensus in the literature regarding the relationship between metformin use and reduced BTC risk.

Metformin is now extensively used as the recommended first-line treatment for type 2 diabetes. Due to the strong association between diabetes and BTC, elucidating the effects of metformin on BTC may provide new ideas for the prevention and management of BTC in clinical practice, particularly in diabetics. Given the lack of consistent evidence, this systematic review and meta-analysis was conducted in order to evaluate the correlation between metformin use and BTC.

## 2. Methods and Materials

This systematic review and meta-analysis followed the Preferred Reporting Items for Systematic Reviews and Meta-analyses (PRISMA 2020) statement [25]. The protocol for this study was not registered. Since all analyses were based on previously published studies, no ethical approval or patient consent was required.

### 2.1. Search Strategy

Without any restrictions, two researchers systematically searched PubMed, Embase, the Cochrane Library, and Web of Science to identify publications relevant to metformin for BTC patients (the search strategy implemented for all databases was from their inception to 31 March 2022). By using Boolean operators, search strategies were developed by combining Medical Subject Headings terms (such as biliary tract neoplasms, cholangiocarcinoma, gallbladder neoplasms, and metformin) with free terms (such as biliary tract cancer, perihilar biliary duct carcinoma, cholangiocellular carcinoma, gallbladder cancer, and dimethylbiguanidine). Any dispute between the two researchers was resolved through negotiation. Otherwise, an experienced expert in this field intervened to solve the problem. The specific search strategies used are provided in the Supplementary Table S1.

### 2.2. Eligibility Criteria and Study Selection

Studies meeting all of the following criteria were eligible for the current study: (1) the study populations are non-specific. Cohort studies investigating endpoints of interest in populations using metformin or case-control studies investigating the effects of metformin in patients with BTC were eligible; (2) studies on the use of metformin before and/or after the diagnosis of BTC; and (3) estimates of the effect of metformin on BTC were conducted for the following endpoints of interest: relative risk of BTC, overall survival (OS), and disease-free survival (DFS) of BTC.

Studies falling under any of the following criteria were excluded: (1) repeated studies, such as conference abstracts that were published in full; (2) case reports or case series with fewer than 20 patients; (3) cross-sectional studies or single-arm studies; (4) non-human studies, such as in vitro studies or animal studies; (5) papers that did not generate primary data, including reviews, meta-analyses, commentaries, or editorials; (6) if most of the enrolled patients were substance withdrawal patients, and (7) our analyses did not identify the intersecting endpoint with the biological indicators or other measures used, or used placebos or control groups in any combination with any intervention.

Based on the inclusion and exclusion criteria, two reviewers independently evaluated the relevant literature. Our first step was to automatically remove duplicate studies and remove irrelevant studies based on their titles and abstracts. Subsequently, we analyzed the full texts of studies we identified as potentially eligible. The reasons for the exclusion of ineligible studies were recorded and cross-checked. The selection of studies was conducted by two independent investigators, and any discrepancies were resolved by consulting a third expert. We downloaded and managed all citations using EndNote X9 software (Thompson ISI ResearchSoft, Clarivate Analytics, Philadelphia, PA, USA).

### 2.3. Data Extraction and Quality Assessment

Data extraction and quality assessment were carried out independently by two reviewers. Pre-designed comprehensive Excel forms were designed to collect the following key data from the included studies: first author, year of publication, country, age, gender, type of BTC, study period and design, population source, sample size in different study groups, analysis mode, adjusted/matched confounders, study endpoints, and follow-up time. By contacting corresponding authors of potential studies, we were able to obtain the essential data when not reported in the original studies.

The quality of the eligible cohort and case–control studies was evaluated using the cohort design version (selection, comparability, and outcome) and the case–control design version (selection, comparability, and exposure) of the Newcastle–Ottawa Scale (NOS), respectively, as independently judged by two researchers. Studies with NOS scores ≥ 7 were considered high-quality studies; otherwise, they were reported as low-quality studies [26].

### 2.4. Statistical Analysis

We conducted a traditional pairwise meta-analysis of the included trials for each existing comparison. Adjusted risk estimates were preferentially extracted for the meta-analysis. Regarding individual studies that assessed the effect of metformin on BTC but lacked the necessary data for quantitative analysis, we provided a narrative description and discussion in the corresponding results section. Hazard risk (HR) was calculated to assess the relationship between metformin and BTC in the adult population. A 95% confidence interval (CI) was used to estimate the scope of the overall parameters [27]. We included the HRs and their 95% CIs as our pooled effects, while such variables must be symmetric and based on a normal distribution, and therefore logarithmic transformation was first performed for the above variables to obtain a normal distribution. Heterogeneity in the included studies was evaluated using Cochran’s Q and the corresponding *p*-value, and a substantial level of heterogeneity was evaluated by the *I*^2^ statistic. The studies were homogeneous if *I*^2^ was <50%, *p* > 0.05, and a fixed effects model was reported. In contrast, if *I*^2^ was ≥50% and *p* < 0.05, a random effects model was reported. A sequence of subgroup analyses was conducted to explain any observed heterogeneity or to explore statistically significant differences between studies; stratified analysis was performed according to participants (restricted to diabetics vs. not restricted to diabetics), median/mean age (<65 years vs. ≥65 years), pathological site, study location (Asia vs. non-Asia), and sample size. Sensitivity analysis was performed by removing each study in turn to re-estimate the effect size and its contribution. Additionally, potential bias in small studies was evaluated using a comparison-adjusted funnel plot, which serves as an intuitive visual instrument for detecting the presence of any dominant types of potential bias, such as publication bias, selective reporting, or other biases. The quantitative Egger’s test was performed to determine whether *p* values were less than 0.05. Otherwise, the trim-and-fill method was used to assess the impact of potentially unpublished studies on the pooled results [28]. All tests were two-tailed; *p* < 0.05 was considered statistically significant. The above sequence of analyses were conducted in STATA, version 16.0 (Stata, Corp., College Station, TX, USA)

## 3. Results

**Figure 1:**
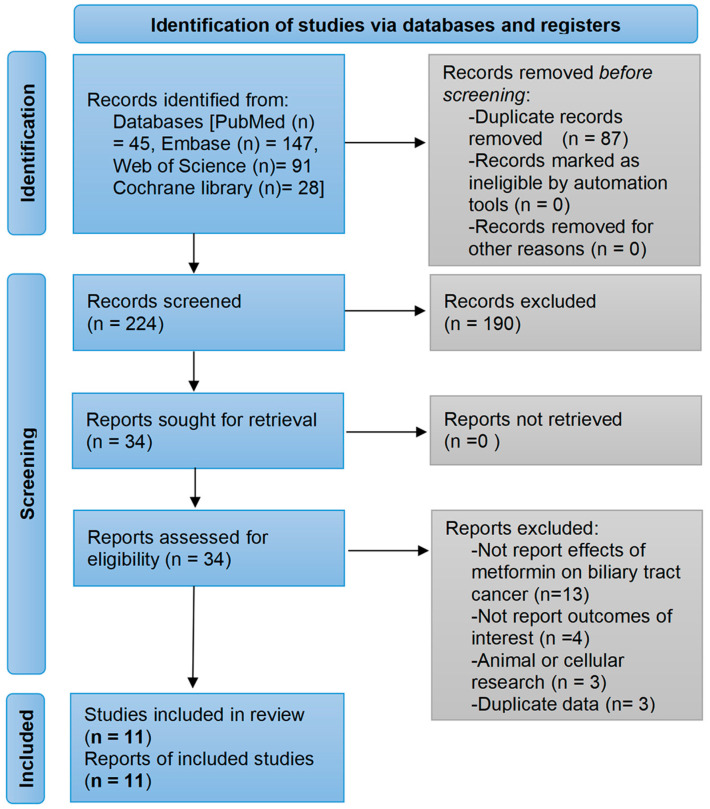
Flow diagram of the study selection process. The search strategies yielded a total of 311 papers of which, 11 studies were finally included based on eligibility criteria.

A total of 311 studies were generated from PubMed, Embase, Web of Science, and the Cochrane library databases by using pre-developed search algorithms. After removing duplicates, 190 clearly ineligible studies were excluded through a review of their titles and abstracts. The remaining 34 studies were read in full, among which 13 studies did not investigate the efficacy of metformin on BTC, 4 studies did not explore risks or prognostic endpoints for BTC, 3 studies were cellular or animal studies, and 3 studies were duplicate reports of the same study. Finally, a total of 11 studies were included in the meta-analysis [21,22,23,24,29,30,31,32,33,34,35]. The detailed process of study selection is presented in Figure 1. Table S2 in the Supplementary file shows the studies included in each pooled analysis.

### 3.1. Study Characteristics

A total of 11 studies involving 24,788,738 participants were finally included: two studies from each of the following countries: Italy, China, and the USA; and one study from each of the following countries: Thailand, Korea, Netherlands, Canada, and Sweden. Seven studies explored the effect of metformin on the risk of BTC [21,22,23,31,32,33,34], five explored its effect on OS [21,24,29,30,35], and two explored its effect on DFS [30,35]. The studies that reported median/mean age all ranged from 60 to 70. With the exception of two case–control studies, the remaining publications were cohort studies. Table 1 summarizes the details of the characteristics of the included studies.

### 3.2. Quality Assessment

Nine cohort studies had NOS scores between 7 and 9, and each case–control study scored 8, indicating a high overall quality level in the published studies (Table 2). The main risk bias came from the unclear follow-up duration.

### 3.3. Association between Metformin Use and Risk of BTC

**Figure 2:**
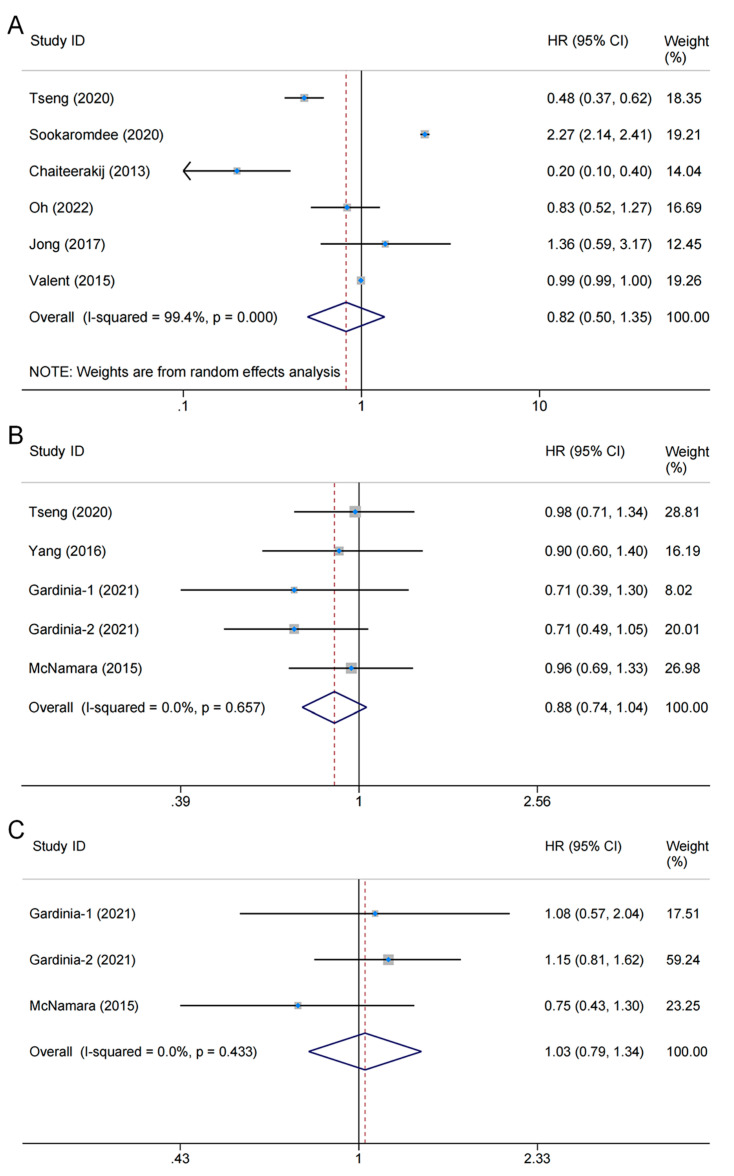
Forest plot of the associations between metformin use and biliary tract cancer: (**A**) risks [21,31,22,23,33,34]; (**B**) overall survival [21,24,30,35]; (**C**) disease-free survival [30,35]. The Study ID column shows the eligible studies included in each endpoint; the HR (95% CI) column is the hazard ratio of the effect of metformin use vs. non-use on outcomes as reported by the eligible studies; Weight column is the weight percentage of each study in the pooled analysis; and the horizontal axis is the scale of effect estimates. The overall pooled results showed no significant differences in biliary tract cancer risk (HR = 0.82, 95% CI: 0.50–1.35, *p* = 0.436), overall survival (HR = 0.88, 95% CI: 0.74–1.04, *p* = 0.135), or disease-free survival (HR = 1.03, 95% CI: 0.79–1.34, *p* = 0.829) between metformin users and non-users. HR: hazard ratio; CI: confidence interval.

A total of seven studies involving 24,786,265 patients explored the association between metformin use and BTC risk, six of which provided sufficient data to be included for the meta-analysis [21,22,23,31,33,34]. Heterogeneity testing showed substantial heterogeneity (*I*^2^ = 99.4%, *p*_heterogeneity_ < 0.001); therefore, a random-effects model was applied. The pooled results showed no correlation between metformin use and BTC risk (HR = 0.82, 95% CI: 0.50–1.35, *p*_overall effect_ = 0.436) (Figure 2A). The other remaining study also found no significant effect of metformin use on risk of BTC [32].

When restricting the study population to people with diabetes, the pooled results from five studies revealed no statistically significant differences between metformin use and BTC (HR = 0.65, 95% CI: 0.39–1.07, *p*_overall effect_ = 0.089) [21,22,23,33,34]. Two studies comparing metformin users with non-metformin users, which included the general population and diabetic patients, did not find a protective effect of metformin against biliary tract carcinogenesis [31,32]. Four studies provided the mean age of the participants, all between 60 and 65 years, and the HR for their combined analysis was 0.56, which also failed to reach statistical significance (95% CI: 0.31–1.02, *p*_overall effect_ = 0.057) [21,22,23,33]. Differences in study location or sample size were not found to have a significant effect on the results (Table 3).

### 3.4. Association between Metformin Use and OS of BTC

Five studies comprising 306,697 participants assessed the effects of metformin on OS in patients with BTC [21,24,29,30,35]. Among them, four studies provided eligible data types for inclusion in the meta-analysis; the level of heterogeneity between studies was subtle (*I*^2^ = 0.0%, *p*_heterogeneity_ = 0.657), and therefore a fixed-effects model was used. The pooled results showed no significant correlation between metformin use and OS (HR = 0.88, 95% CI: 0.74–1.04, *p*_overall effect_ = 0.135) [21,24,30,35] (Figure 2B). The other remaining study found similar results (95% CI: −17.05–0.375; *p*_overall effect_ = 0.061) [29].

In two studies, participants were restricted to those with diabetes, and the pooled HR was 0.95 (95% CI: 0.74–1.23, *p* = 0.695), with slight heterogeneity (*I*^2^ = 0.0%, *p*_heterogeneity_ = 0.753) [21,24]. Interestingly, in contrast to the analysis of risk effects, metformin users were found to have a tendency toward longer survival in the general population, while a significant difference between metformin and BTC was not observed (HR = 0.82, 95% CI: 0.65–1.04, *p*_overall effect_ = 0.097) [29,30]. There was also no statistical significance in non-Asian populations (HR = 0.84, 95% CI: 0.69–1.03, *p*_overall effect_ = 0.091) [24,30,35]. There were no findings that varied with age, site of pathology, or sample size (Table 3). Interestingly, Gardini et al. reported significantly improved OS for metformin users compared to never users in patients with advanced disease receiving chemotherapy (HR = 0.70, 95% CI: 0.52–0.93, *p* _overall effect_ = 0.016); however, the level of significance decreased after adjusting for potential confounders (HR = 0.71, 95% CI: 0.49–1.05, *p* _overall effect_ = 0.08) [30]. In another study, Wu et al. demonstrated that metformin could prolong OS in patients with advanced extrahepatic cholangiocarcinoma treated with drainage (median survival time: 121 days for metformin group vs. 116 days for control group, *p* _overall effect_ = 0.026) [29].

### 3.5. Association between Metformin Use and DFS of BTC

Two studies comprising 1501 patients explored the effect of metformin on DFS [30,35]. Notably, the control group was not restricted to diabetic patients. The heterogeneity was subtle (*I*^2^ = 0.0%, *p*_heterogeneity_ = 0.433), and combined results using a fixed-effects model revealed that metformin use did not significantly affect DFS (HR = 1.03, 95% CI: 0.79–1.34, *p*_overall effect_ = 0.829) (Figure 2C).

### 3.6. Publication Bias

**Figure 3:**
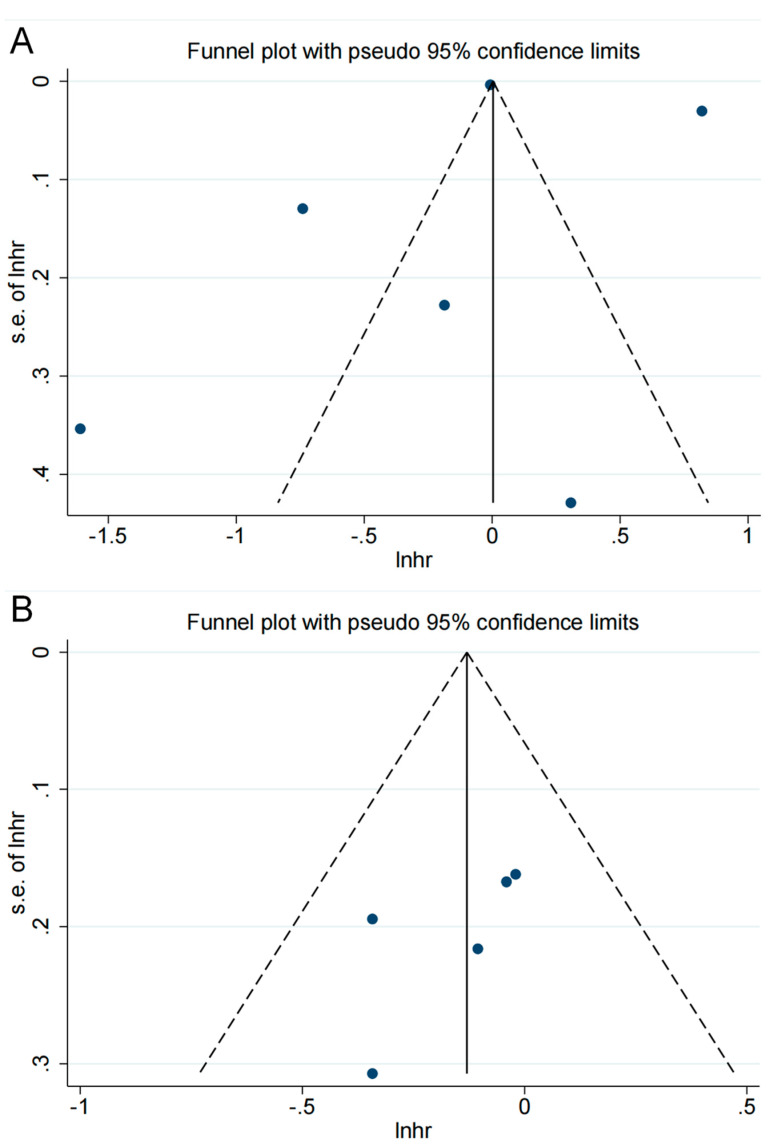
The funnel plot for publication bias assessment: (**A**) in risk analysis; (**B**) in overall survival analysis. The horizontal axis is the scale of the natural logarithm of the effect size, and the vertical axis is its standard error. The solid line perpendicular to the horizontal axis is the natural logarithm of the pooled effect size using the fixed-effects model; the dashed line is the pseudo-95% confidence interval lines. The dots in the graph represent the individual studies included. Studies with larger sample sizes are usually distributed at the top of the graph, while studies with smaller samples are distributed at the bottom. Ideally, studies are evenly distributed on both sides of the solid line. However, due to the small number of included studies, we simply examined whether there was a significant difference in the number of studies on either side of the solid line and then combined this with quantitative Egger’s and Begg’s tests to assess the risk of publication bias. Roughly, no significant asymmetric distribution was observed.

The endpoints for five or more cohorts were examined for publication bias. No clear asymmetry was found in the funnel plots (Figure 3); *p*-values for both Begg’s and Egger’s tests were greater than 0.05 (0.452 for Begg and 0.670 for Egger in the risk analysis; 0.221 for Begg and 0.234 for Egger in the survival analysis), demonstrating that there was no evidence of risk of publication bias.

### 3.7. Sensitivity Analysis

**Figure 4:**
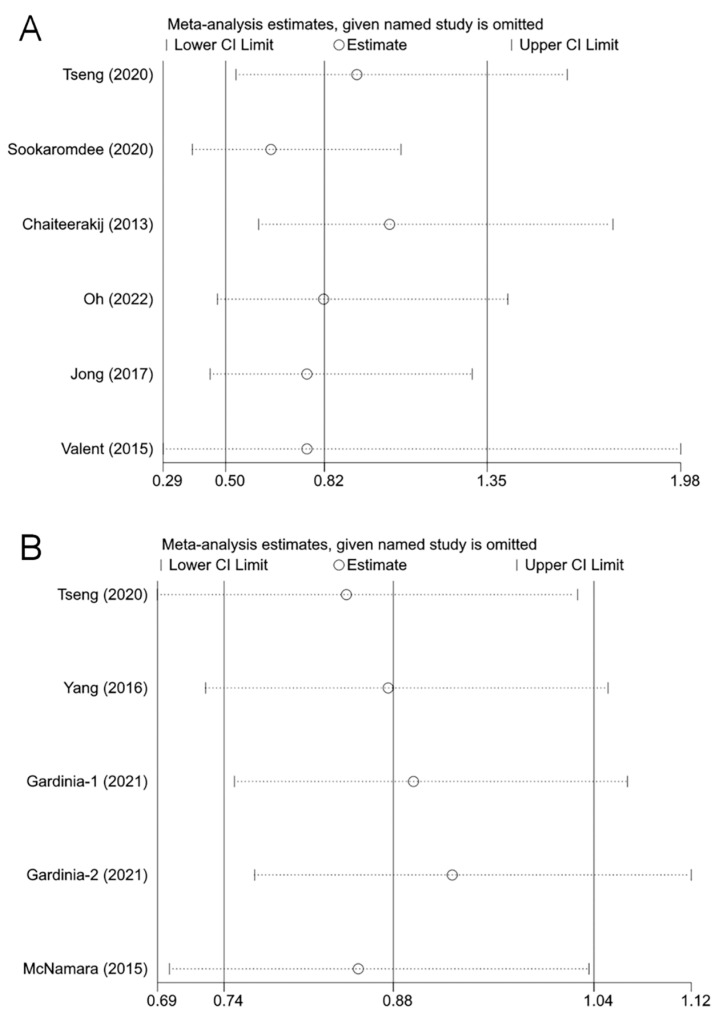
The effects of the individual studies on the pooled effect size: (**A**) biliary tract cancer risk [21,31,22,23,33,34]; (**B**) overall survival [21,24,30,35]. The horizontal axis is the scale of effect estimates. On the left side of the vertical axis are the studies that were excluded in turn; the corresponding effect estimates and confidence intervals on the right side are the results of the meta-analysis for all studies except for the excluded study in that row. A study was considered to have an excessive influence on the pooled results if the result of the re-analysis after excluding that study exceeded the confidence intervals of the overall meta-analysis. The effect sizes re-estimated after removing each study in turn did not change significantly, indicating that the current results are relatively stable.

Sensitivity analysis was performed by removing one study at a time and re-merging the remaining ones. There was no significant change in the pooled results and individual studies had little effect on the combined results, indicating that the current pooled results are relatively stable (Figure 4).

## 4. Discussion

This is the first systematic review and meta-analysis to synthesize the current evidence on the association between metformin use and the risk and prognosis of BTC. The current evidence suggests that, in general, the effect of metformin use on BTC incidence and survival lacks statistical significance. The results need to be interpreted cautiously due to certain limitations in our study, and more high-quality relevant research needs to be performed for the establishment of a comprehensive evidence base to verify the association between metformin and BTC.

Notably, there was significant heterogeneity in the analysis of the effect of metformin on BTC risk; even in subgroup analyses based on main characteristics, the heterogeneity was still not significantly reduced. By reviewing the study characteristics and design, we considered that heterogeneity might be primarily derived from the selection of the participants and the definition of metformin users. Prior studies have identified an increased risk of BTC in diabetic patients [10,11,36]. Therefore, in studies by Sookaromdee et al. and Bonilla et al., it was expected that the incidence of BTC would not be reduced in patients using metformin compared to the controls, including the general population, as using metformin tends to represent a diagnosis of diabetes [31,32]. Instead, Sookaromdee et al. discovered a slight reduction in cancer risk in patients with diabetes treated with metformin in areas where bile duct cancer is endemic (odds ratio: 2.27 vs. 2.31, diabetics using metformin and general diabetics, respectively) [31]. Chaiteerakij et al. reported that metformin use can reduce the risk of ICC by 60% among diabetic patients [22], comparable to the reductions seen in other cancers (50–85%) including breast cancer, hepatocellular carcinoma, colorectal, breast, and lung cancer [37,38,39,40,41,42]. The antitumor effects of metformin on breast and prostate cancer cells have been demonstrated in in vivo and in vitro experiments, since rapamycin/ribosomal protein S6 kinase beta-1 (mTOR/S6K1) activity is suppressed by adenosine monophosphate-activated protein kinase (AMPK) activation [43,44]. However, it is currently unknown whether metformin affects malignant cholangiocytes as well.

Another important potential source of heterogeneity was the inconsistent definition of a metformin user. In the study conducted by Oh et al., metformin users were defined as those who had been taking oral metformin consistently for more than 90 days, while other individuals with diabetes were used as controls [23]; in the study by Tseng et al., patients with a history of metformin use were considered to be metformin users, and controls were diabetics who had never used metformin [21]; the study by Jong et al. analyzed the difference between current metformin users and current non-metformin non-insulin antidiabetic drug users (current users were defined as patients who had received the target drug within 90 days prior to the start of the follow-up interval) [33]. Four other studies did not define a metformin user [22,31,32,34]. Only Oh et al. clearly defined the dose and duration of metformin use [23]. A stratified analysis of cumulative metformin doses at low, medium, and high levels was performed by Jong et al., and no significant differences in endpoints were observed between the strata [33]. Interestingly, a stratified analysis of the cumulative duration of metformin use by Tseng et al. determined that a longer cumulative duration of metformin treatment resulted in a greater reduction in the risk of subsequent development of BTC, with an approximately 90% reduction in the incidence of BTC in patients treated cumulatively for more than 46 months and no risk reduction in patients treated for less than 22 months, which appears to imply a time-dependent response pattern [21]. Another interesting study by Jong et al. investigated the effect of metformin with other antidiabetic drugs on outcomes; regrettably, no meaningful results were achieved owing to the relatively small sample size and lack of statistical power [33]. Tseng et al. also explored the interaction of several variables with metformin, including demographic data, occupation, living region, comorbidities, diabetes-related complications, antidiabetic drugs, potential risk factors of cancer, and medications, with only *Helicobacter pylori* infection showing significance [21]. In addition, differences in the types of diabetes and BTC included in the studies were potential sources of heterogeneity. However, due to a scarcity of eligible studies, no reliable inferences could be made. BTC is a highly malignant tumor, and if its early prevention with metformin is effective, especially in those at risk, such as diabetics and the elderly, the burden of this life-threatening cancer could be reduced [3]. In several studies, metformin has been shown to protect diabetic patients against ICC compared to those not treated with metformin. This may be due to differences in the baseline characteristics of diabetic patients treated with metformin., i.e., diabetes patients with shorter duration or less severe diabetes are less likely to develop cancer, as metformin is a marker of shorter duration or less severe diabetes [22]. Future studies should examine the effect of severity of diabetes on BTC risk, and whether mild diabetes patients have a lower risk of BTC than those with more severe diabetes.

In contrast to the risk analysis, no substantial statistical heterogeneity was observed in the pooled analysis of the effect of metformin on survival. However, differences in the clinical characteristics of the included studies, such as the factors discussed above, may still impact the effect of metformin on BTC. Currently, no randomized controlled studies with metformin as a variable have been conducted. In fact, the studies included for quantitative analysis were more inclined to act as a correlational analysis rather than to explore the therapeutic effects of metformin. Only the study by Wu et al. can be considered as a clinical trial exploring metformin for BTC, but not using metformin was also the only treatment or it was used in combination with a specific regimen [29]; they instructed the trial group to take 0.5 g of metformin thrice daily and found a significant correlation between metformin use and prolonged OS in patients treated with drainage. In addition, they established that age had no impact on the effect of metformin, whereas the timing and duration of metformin use had a significant effect on the outcomes; patients who received metformin following the diagnosis of advanced cholangiocarcinoma and had a treatment course greater than three months had superior OS [29]. A study by Gardinia et al. found similar results, with metformin use associated with prolonged OS in patients with advanced disease receiving chemotherapy; in the multivariable analysis, this correlation was present in patients taking metformin after the initiation of chemotherapy, while there was no difference among patients receiving metformin before chemotherapy compared to never users [30]. This difference may be related to the lack of sensitivity to metformin in patients with early-stage BTC. There are no studies showing that metformin improves DFS in a certain setting. Overall, metformin appears to have the potential to improve survival in advanced settings, and this potential is related to the timing of therapy initiation and its duration. Metformin has been shown to increase the antitumor activity of cisplatin, a commonly used drug in BTC, through the AMPK–mTOR, PI3K/AKT/ERK, and oxidative stress-mediated mitochondrial pathways, resulting in a synergistic effect [45,46,47]. Moreover, metformin also sensitizes cholangiocarcinoma cells to chemotherapeutic agents such as sorafenib, 5-fluorouracil, and arsenic trioxide by regulating the AMPK/mTOR/HIF-1α/MRP1 pathway and ERK [48,49]. Thus, the chemotherapeutic effects of metformin in BTC and its combination with chemotherapeutic agents warrant further exploration in future clinical settings.

Several pre-clinical studies have attempted to investigate the effects of metformin on BTC, and metformin was found to affect the development and progression of BTC through several mechanisms. The Warburg effect refers to alterations in cellular metabolism from oxidative phosphorylation to glycolysis as a result of tumor development and proliferation. Tang et al. demonstrated in vitro that metformin can reverse the Warburg effect by inhibiting the expression of lactate dehydrogenase-A, thereby impacting the metabolism of cancer cells [50]. Upregulation of the mammalian target of rapamycin (mTOR) is often observed in BTCs; however, metformin can directly activate the AMP-activated protein kinase (AMPK) signaling pathway, leading to inhibition of the mTOR signaling pathway, thereby downregulating protein synthesis, cell proliferation, and tumor angiogenesis [51,52,53]. Metformin also exerts its anti-proliferative effects through cell cycle regulators, including the downregulation of cyclin D1, a key protein required for cell cycle G_1_, and the activation of caspase-3, thereby blocking the transition from G_0_ to G_1_ cell cycle [20,54]. In addition, the association of diabetes and inflammation with BTC has been widely mentioned, and metformin may also inhibit cancer development by lowering blood sugar, improving insulin resistance, reducing metabolic disorders such as dyslipidemia, and exerting anti-inflammatory effects [10,55,56]. Indeed, metformin has been shown to reduce the risk and improve the prognosis of many other cancers, such as lung cancers, colorectal cancers, pancreatic cancers, gynecology-related cancers, prostate cancers, urological cancers, thyroid cancers, skin cancers, and lymphomas. Thus, the anti-cancer effects of metformin may involve some common pathophysiological mechanisms associated with the development and progression of other cancers [57,58,59,60,61,62,63,64,65,66].

As far as we know, this is the first attempt to summarize the available evidence to evaluate the relationship between metformin use and BTC-related outcomes. To prevent the omission of studies, potential studies were obtained by screening references of similar studies and by manual searches during the study selection process. We used the prospective or retrospective collection of data from various sources including health care records and hospital records, and the method of data collection was comparable between the exposure/case and control groups. Our study had a large sample size, although despite the battery of analyses, the association between metformin and BTC was not confirmed. To summarize, our study contributes to the latest evidence by providing a detailed summary of the evidence regarding the association between metformin and BTC. As a result of this study, policymakers, clinicians, or caregivers may be able to make better decisions and navigate the direction of clinical decision making, therefore facilitating future research and clinical applications in the future. There are several limitations to this study that need to be considered. First, there were relatively few eligible studies per endpoint and in certain populations; although population-based studies provide greater sample sizes, further investigation is still required to validate the effect of metformin on BTC in different geographical areas and populations with different characteristics. Second, registry studies identifying patients by diagnostic codes are subject to information bias, such as inevitable misdiagnosis and missed diagnoses. Third, the study designs in the risk analysis were retrospective. Therefore, some potentially confounding bias data, including the impact of physical activity, dietary habits, family history, co-morbidities, and diabetes severity, were not adjusted for. Fourth, most studies lacked clear definitions of metformin users, such as the specific doses and regimens.

## 5. Conclusions

There is substantial heterogeneity in the current studies exploring the association between metformin and BTC, and the present study suggests that metformin may not have the capacity to inhibit the risk of carcinogenesis and exert chemotherapeutic effects. The results need to be interpreted cautiously due to certain limitations in our study. Future prospective studies are needed to further validate and elucidate the effects of metformin on different patient characteristics, particularly in non-diabetic patients, and the optimal timing, dose, and duration of application.

## Figures and Tables

**Table 1:** Characteristics of studies investigating the effects of metformin on biliary tract cancer.

Study	Country	Period	Study Design	Mean/Median Age (Years)	Data Source	N (Metformin or Case Group/Non-Metformin or Control Group)	Pathological Type	Analysis Mode	Adjustment/Matching Confounder	Study Endpoints	Follow-Up
Tseng, 2020 [21]	Taiwan, China	1999–2011	Cohort study	Metformin group: 63.6; non-metformin group: 61.4	Health care records	304,224 (16,229/287,995)	NR	Multi	Demographic data, occupation, region of residence, major comorbidities, diabetes-related complications, antidiabetic drugs, potential risk factors of cancer, medications that are commonly used in diabetes patients or may affect cancer risk, follow-up duration	OS, incidence risk	>6 months
Sookaromdee, 2020 [31]	Thailand	NR	Case–control	NR	Hospital records	18,547,869 (NR)	CCA	NR	NR	Incidence risk	NR
Wu, 2021 [29]	China	2015–2021	Cohort study	Median ≥ 60	Hospital records	722 (133/589)	CCA	NR	Sex, age, ethnicity, and place of residence	OS	6 months
Yang, 2016 [24]	USA	2001–2012	Cohort study	Mean 68	Hospital records	250 (49/165) *	CCA	Multi	Age, sex, and variables with a *p* ≤ 0.10 in the univariate analysis	OS	NR
Gardini, 2021 [30]	Italy	2005–2020	Cohort study	NR	Hospital records	706 (57/643) ^	557 CCA, 149 GC	Uni	Eastern Cooperative Oncology Group performance status, carbohydrate antigen 19-9, carcinoembryonic antigen, platinum plus gemcitabine therapy versus other therapy, and primary tumor site	OS, DFS	NR
Chaiteerakij, 2013 [22]	USA	2000–2010	Case–control	Case group: 61.2; control group: 61.6	Hospital records	1206 (612/594)	ICCA	Multi	Age, sex, ethnicity, and place of residence	Incidence risk	NR
Oh, 2020 [23]	Korea	2011–2015	Cohort study	Mean 60.1	Health care records	66,627 (29,974/36,653)	BTC	Multi	Age, sex, income level, place of residence, hypertension, coronary artery disease, cerebrovascular disease, psychobehavioral disorder, musculoskeletal disorders, chronic kidney disease, dyslipidemia, anemia, chronic obstructive pulmonary disease, arrhythmia, liver cirrhosis, receipt of surgery, total number of hospital visit days, and use of other antidiabetic medications (sulfonylureas, alpha-glucosidase inhibitors, thiazolidinediones, and insulin)	Incidence risk	NR
Jong, 2017 [33]	Netherlands	1998–2011	Cohort study	Metformin group: mean 63.5; non-metformin: 67.0	Hospital records	57,114 (37,215/19,899)	GC	Multi	Age, sex, use of statins, history of hospitalization	Incidence risk	Mean 4.9 years
Valent, 2015 [34]	Italy	2002–2014	Cohort study	NR	Health care records	109,225 (NR)	GC	Multi	Sex, age, time when prescription of each drug started (time-dependent variable), and total number of prescriptions of all the other drugs	Incidence risk	NR
Bonilla, 2019 [32]	Swedish	NR	Cohort study	NR	Prescribed drug records	5,700,000 (NR)	BTC	NR	NR	Incidence risk	NR
McNamara, 2015 [35]	Canada	1987–2013	Cohort study	Median 65.7	NR	795 (81/714)	BTC	NR	NR	OS, DFS	NR

Abbreviations: BTC, biliary tract cancer; CCA, cholangiocarcinoma; DFS, disease-free survival; GC, gallbladder cancer; ICCA, intrahepatic cholangiocarcinoma; NR, not reported; OS, overall survival. * There may be cases of missed visits. ^ No data for six participants.

**Table 2:** The quality assessment of included studies.

Study (Cohort)	Representativeness of Exposed Cohort	Selection of Non-Exposed Cohort	Ascertainment of Exposure	Outcome not Present before Study	Comparability	Assessment of Outcome	Follow-Up Long Enough	Adequacy of Follow Up *	Quality Score
Tseng, 2020 [21]	★	★	★	★	★★	★	☆	★	8
Wu, 2021 [29]	★	★	★	★	★☆	★	☆	★	7
Yang, 2016 [24]	★	★	★	★	★★	★	☆	★	8
Gardini, 2021 [30]	★	★	★	★	★☆	★	☆	★	7
Oh, 2020 [23]	★	★	★	★	★★	★	☆	★	8
Jong, 2017 [33]	★	★	★	★	★★	★	★	★	9
Valent, 2015 [34]	★	★	★	★	★★	★	☆	★	8
Bonilla, 2019 [32]	★	★	★	★	★☆	★	☆	★	7
McNamara, 2015 [35]	★	★	★	★	★☆	★	☆	★	7
**Study (Case–control)**	**Case Definition**	**Representativeness of the Cases**	**Selection of Controls**	**Definition of Controls**	**Comparability**	**Ascertainment of Exposure**	**Same Method of Ascertainment**	**Non-Response Rate**	**Quality Score**
Sookaromdee, 2020 [31]	★	★	★	★	★☆	★	★	★	8
Chaiteerakij, 2013 [22]	★	★	★	★	★☆	★	★	★	8

* Median follow-up of more than three years or maximum follow-up of more than 5 years was considered enough.

**Table 3:** Stratified analysis of the association between metformin and the risk and prognosis of biliary tract cancer.

Subgroups	Studies	HR (95%CI)	*p _Z_*	Heterogeneity (*I*^2^, *p_H_*)	Effects Model
Risk of biliary tract cancer	6	0.82 (0.50–1.35)	0.436	99.4%, <0.001	Random
*Participants*					
Diabetics	5	0.65 (0.39–1.07)	0.089	92.5%, <0.001	Random
General population	1	2.27 (2.14–2.41)	<0.001	NA	NA
*Median/mean age*					
<65 years	4	0.56 (0.31–1.02)	0.057	82.3%, 0.001	Random
≥65 years	0	NA	NA	NA	NA
*Pathological site*					
Cholangiocarcinoma	2	0.69 (0.06–7.47)	0.761	97.9%, <0.001	Random
Gallbladder cancer	1	0.993 (0.986–1.000)	0.051	NA	NA
*Study location*					
Asia	3	0.97 (0.31–3.08)	0.960	98.7%, <0.001	Random
non-Asia	3	0.65 (0.24–1.80)	0.409	90.5%, <0.001	Random
*Sample size*					
<100,000	3	0.60 (0.22–1.70)	0.339	86.8%, <0.001	Random
≥100,000	3	1.04 (0.53–2.01)	0.916	99.7%, <0.001	Random
Overall survival	4	0.88 (0.74–1.04)	0.135	0.0%, 0.657	Fixed
*Participants*					
Diabetics	2	0.95 (0.74–1.23)	0.695	0.0%, 0.753	Fixed
General population	2	0.82 (0.65–1.04)	0.097	0.0%, 0.437	Fixed
*Median/mean age*					
<65 years	1	0.98 (0.71–1.34)	0.901	NA	NA
≥65 years	2	0.94 (0.72–1.21)	0.623	0.0%, 0.813	Fixed
*Pathological site*					
Cholangiocarcinoma	1	0.90 (0.60–1.40)	0.70	NA	NA
*Study location*					
Asia	1	0.98 (0.71–1.34)	0.901	NA	NA
Non-Asia	3	0.84 (0.69–1.03)	0.091	0.0%, 0.618	Fixed
*Sample size*					
<300	2	0.83 (0.59–1.18)	0.298	0.0%, 0.528	Fixed
≥300	3	0.89 (0.73–1.09)	0.260	0.0%, 0.385	Fixed
Disease-free survival	2	1.03 (0.79–1.34)	0.829	0.0%, 0.433	Fixed

Abbreviations: HR, hazard risk; OS, overall survival; DFS, disease-free survival; NA, not applicable.

## Data Availability

Please see the Supplementary Materials.

## Supplementary Materials

The following supporting information can be downloaded at: https://www.mdpi.com/article/10.3390/medicina59020298/s1, Table S1: Search strategies for each database; Table S2: Studies included in each meta-analysis.

## References

[1] Valle J.W., Kelley R.K., Nervi B., Oh D.-Y., Zhu A.X. (2021). Biliary tract cancer. Lancet.

[2] Nagtegaal I.D., Odze R.D., Klimstra D., Paradis V., Rugge M., Schirmacher P., Washington K.M., Carneiro F., Cree I.A., WHO Classification of Tumours Editorial Board (2020). The 2019 WHO classification of tumours of the digestive system. Histopathology.

[3] Torre L.A., Siegel R.L., Islami F., Bray F., Jemal A. (2018). Worldwide Burden of and Trends in Mortality from Gallbladder and Other Biliary Tract Cancers. Clin. Gastroenterol. Hepatol..

[4] Ferlay J., Soerjomataram I., Dikshit R., Eser S., Mathers C., Rebelo M., Parkin D.M., Forman D., Bray F. (2015). Cancer incidence and mortality worldwide: Sources, methods and major patterns in GLOBOCAN 2012. Int. J. Cancer.

[5] Zhao D.Y., Lim K.-H. (2017). Current biologics for treatment of biliary tract cancers. J. Gastrointest. Oncol..

[6] Lamarca A., Edeline J., Goyal L. (2022). How I treat biliary tract cancer. ESMO Open.

[7] Morizane C., Ueno M., Ikeda M., Okusaka T., Ishii H., Furuse J. (2018). New developments in systemic therapy for advanced biliary tract cancer. Jpn. J. Clin. Oncol..

[8] Ciardiello D., Maiorano B.A., Parente P., Rodriquenz M.G., Latiano T.P., Chiarazzo C., Pazienza V., Guerrera L.P., Amoruso B., Normanno N. (2022). Immunotherapy for Biliary Tract Cancer in the Era of Precision Medicine: Current Knowledge and Future Perspectives. Int. J. Mol. Sci..

[9] Chakrabarti S., Kamgar M., Mahipal A. (2020). Targeted Therapies in Advanced Biliary Tract Cancer: An Evolving Paradigm. Cancers.

[10] Gu J., Yan S., Wang B., Shen F., Cao H., Fan J., Wang Y. (2015). Type 2 diabetes mellitus and risk of gallbladder cancer: A systematic review and meta-analysis of observational studies. Diabetes Metab. Res. Rev..

[11] Tsilidis K.K., Kasimis J.C., Lopez D.S., Ntzani E.E., Ioannidis J.P.A. (2015). Type 2 diabetes and cancer: Umbrella review of meta-analyses of observational studies. BMJ.

[12] Jing C., Wang Z., Fu X. (2020). Effect of diabetes mellitus on survival in patients with gallbladder Cancer: A systematic review and meta-analysis. BMC Cancer.

[13] Roderburg C., Loosen S.H., Hoyer L., Luedde T., Kostev K. (2022). Prevalence of diabetes mellitus among 80,193 gastrointestinal cancer patients in five European and three Asian countries. J. Cancer Res. Clin. Oncol..

[14] Gurney J., Stanley J., Teng A., Krebs J., Koea J., Lao C., Lawrenson R., Meredith I., Sika-Paotonu D., Sarfati D. (2022). Cancer and diabetes co-occurrence: A national study with 44 million person-years of follow-up. PLoS ONE.

[15] Sanchez-Rangel E., Inzucchi S.E. (2017). Metformin: Clinical use in type 2 diabetes. Diabetologia.

[16] Lv Z., Guo Y. (2020). Metformin and Its Benefits for Various Diseases. Front. Endocrinol..

[17] Morales D.R., Morris A.D. (2015). Metformin in Cancer Treatment and Prevention. Annu. Rev. Med..

[18] Di Matteo S., Nevi L., Overi D., Landolina N., Faccioli J., Giulitti F., Napoletano C., Oddi A., Marziani A.M., Costantini D. (2021). Metformin exerts anti-cancerogenic effects and reverses epithelial-to-mesenchymal transition trait in primary human intrahepatic cholangiocarcinoma cells. Sci. Rep..

[19] Yamashita T., Kato K., Fujihara S., Iwama H., Morishita A., Yamana H., Kobayashi K., Kamada H., Chiyo T., Kobara H. (2020). Anti-diabetic drug metformin inhibits cell proliferation and tumor growth in gallbladder cancer via G0/G1 cell cycle arrest. Anti-Cancer Drugs.

[20] Zhu H.-Q., Ma J.-B., Song X., Gao H.-J., Ma C.-Q., Chang H., Li H.-G., Liu F.-F., Lu J., Zhou X. (2016). Metformin potentiates the anticancer activities of gemcitabine and cisplatin against cholangiocarcinoma cells in vitro and in vivo. Oncol. Rep..

[21] Tseng C.-H. (2020). Metformin and Biliary Tract Cancer in Patients With Type 2 Diabetes. Front. Oncol..

[22] Chaiteerakij R., Yang J.D., Harmsen W.S., Slettedahl S., Mettler T.A., Fredericksen Z.S., Kim W.R., Gores G.J., Roberts R.O., Olson J.E. (2012). Risk factors for intrahepatic cholangiocarcinoma: Association between metformin use and reduced cancer risk. Hepatology.

[23] Oh T.K., Song I.-A. (2020). Metformin Use and the Risk of Cancer in Patients with Diabetes: A Nationwide Sample Cohort Study. Cancer Prev. Res..

[24] Yang Z., Zhang X., Roberts R.O., Roberts L.R., Chaiteerakij R. (2016). Metformin does not improve survival of cholangiocarcinoma patients with diabetes. Hepatology.

[25] Page M.J., McKenzie J.E., Bossuyt P.M., Boutron I., Hoffmann T.C., Mulrow C.D., Shamseer L., Tetzlaff J.M., Akl E.A., Brennan S.E. (2021). The PRISMA 2020 statement: An updated guideline for reporting systematic reviews. Syst. Rev..

[26] Wells G.A., Shea B., O’Connell D., Pereson J., Welch V., Losos M., Tugwell P. The Newcastle–Ottawa Scale (NOS) for Assessing the Quality of Nonrandomised Studies in Meta-Analyses. http://www.ohri.ca/programs/clinical_epidemiology/oxford.asp.

[27] DerSimonian R., Laird N. (1986). Meta-analysis in clinical trials. Control Clin. Trials.

[28] Duval S., Tweedie R. (2000). Trim and Fill: A Simple Funnel-Plot-Based Method of Testing and Adjusting for Publication Bias in Meta-Analysis. Biometrics.

[29] Wu J., Zhou Y., Wang G. (2021). Metformin Use and Survival in Patients with Advanced Extrahepatic Cholangiocarcinoma: A Single-Center Cohort Study in Fuyang, China. Gastroenterol. Res. Pract..

[30] Casadei-Gardini A., Filippi R., Rimini M., Rapposelli I.G., Fornaro L., Silvestris N., Aldrighetti L., Aimar G., Rovesti G., Bartolini G. (2021). Effects of Metformin and Vitamin D on Clinical Outcome in Cholangiocarcinoma Patients. Oncology.

[31] Sookaromdee P., Wiwanitkit V. (2020). Decreased risk of cholangiocarcinoma in diabetic patients treated with metformin. J. Cancer Res. Ther..

[32] Bonilla L.M., Schleck C., Harmsen W., Therneau T., Sadr-Azodi O., Roberts L.R., Brusselaers N. (2019). 3437 Associations of aspirin, non-aspirin NSAIDs, statins, and metformin with risk of biliary cancer: A Swedish population-based cohort study. J. Clin. Transl. Sci..

[33] De Jong R.G., Burden A.M., de Kort S., van Herk-Sukel M.P., Vissers P.A., Janssen P.K., Haak H.R., Masclee A.A., de Vries F., Janssen-Heijnen M.L. (2017). No Decreased Risk of Gastrointestinal Cancers in Users of Metformin in The Netherlands; A Time-Varying Analysis of Metformin Exposure. Cancer Prev. Res..

[34] Valent F. (2015). Diabetes mellitus and cancer of the digestive organs: An Italian population-based cohort study. J. Diabetes Its Complicat..

[35] McNamara M.G., Aneja P., Le L.W., Horgan A.M., McKeever E., Knox J.J. (2014). Effects of statin, aspirin, or metformin use on recurrence free and overall survival in patients with biliary tract cancer (BTC). J. Clin. Oncol..

[36] Park J.-H., Hong J.Y., Park Y.S., Kang G., Han K., Park J.O. (2021). Association of prediabetes, diabetes, and diabetes duration with biliary tract cancer risk: A nationwide cohort study. Metabolism.

[37] Lee J.H., Kim T.I., Jeon S.M., Hong S.P., Cheon J.H., Kim W.H. (2011). The effects of metformin on the survival of colorectal cancer patients with diabetes mellitus. Int. J. Cancer.

[38] Lai S.-W., Chen P.-C., Liao K.-F., Muo C.-H., Lin C.-C., Sung F.-C. (2012). Risk of Hepatocellular Carcinoma in Diabetic Patients and Risk Reduction Associated With Anti-Diabetic Therapy: A Population-Based Cohort Study. Am. J. Gastroenterol..

[39] Lee M.-S., Hsu C.-C., Wahlqvist M.L., Tsai H.-N., Chang Y.-H., Huang Y.-C. (2011). Type 2 diabetes increases and metformin reduces total, colorectal, liver and pancreatic cancer incidences in Taiwanese: A representative population prospective cohort study of 800,000 individuals. BMC Cancer.

[40] Hassan M.M., Curley S.A., Dalia M.M., Kaseb A., Davila M., Abdalla E.K., Javle M., Bs D.M.M., Lozano R.D., Abbruzzese J.L. (2010). Association of diabetes duration and diabetes treatment with the risk of hepatocellular carcinoma. Cancer.

[41] Donadon V., Balbi M., Mas M.D., Casarin P., Zanette G. (2010). Metformin and reduced risk of hepatocellular carcinoma in diabetic patients with chronic liver disease. Liver Int..

[42] Li D., Yeung S.J., Hassan M.M., Konopleva M., Abbruzzese J.L. (2009). Antidiabetic Therapies Affect Risk of Pancreatic Cancer. Gastroenterology.

[43] Ben Sahra I., Laurent K., Loubat A., Giorgetti-Peraldi S., Colosetti P., Auberger P., Tanti J.F., Le Marchand-Brustel Y., Bost F. (2008). The antidiabetic drug metformin exerts an antitumoral effect in vitro and in vivo through a decrease of cyclin D1 level. Oncogene.

[44] Zakikhani M., Dowling R., Fantus I.G., Sonenberg N., Pollak M. (2006). Metformin Is an AMP Kinase–Dependent Growth Inhibitor for Breast Cancer Cells. Cancer Res.

[45] Wandee J., Prawan A., Senggunprai L., Kongpetch S., Kukongviriyapan V. (2018). Metformin sensitizes cholangiocarcinoma cell to cisplatin-induced cytotoxicity through oxidative stress mediated mitochondrial pathway. Life Sci..

[46] Wandee J., Prawan A., Senggunprai L., Kongpetch S., Tusskorn O., Kukongviriyapan V. (2018). Metformin enhances cisplatin induced inhibition of cholangiocarcinoma cells via AMPK-mTOR pathway. Life Sci..

[47] Bi T., Zhu A., Yang X., Qiao H., Tang J., Liu Y., Lv R. (2017). Metformin synergistically enhances antitumor activity of cisplatin in gallbladder cancer via the PI3K/AKT/ERK pathway. Cytotechnology.

[48] Ling S., Xie H., Yang F., Shan Q., Dai H., Zhuo J., Wei X., Song P., Zhou L., Xu X. (2017). Metformin potentiates the effect of arsenic trioxide suppressing intrahepatic cholangiocarcinoma: Roles of p38 MAPK, ERK3, and mTORC1. J. Hematol. Oncol..

[49] Ling S., Feng T., Ke Q., Fan N., Li L., Li Z., Dong C., Wang C., Xu F., Li Y. (2014). Metformin inhibits proliferation and enhances chemosensitivity of intrahepatic cholangiocarcinoma cell lines. Oncol. Rep..

[50] Tang D., Xu L., Zhang M., Dorfman R.G., Pan Y., Zhou Q., Zhou L., Wang Y., Li Y., Yin Y. (2018). Metformin facilitates BG45-induced apoptosis via an anti-Warburg effect in cholangiocarcinoma cells. Oncol. Rep..

[51] Zhang J., Hang C., Jiang T., Yi S., Shao W., Li W., Lin D. (2020). Nuclear Magnetic Resonance-Based Metabolomic Analysis of the Anticancer Effect of Metformin Treatment on Cholangiocarcinoma Cells. Front. Oncol..

[52] Maemura K., Natsugoe S., Takao S. (2014). Molecular mechanism of cholangiocarcinoma carcinogenesis. J. Hepato Biliary Pancreat. Sci..

[53] Viollet B., Guigas B., Garcia N.S., Leclerc J., Foretz M., Andreelli F. (2012). Cellular and molecular mechanisms of metformin: An overview. Clin. Sci..

[54] Fujimori T., Kato K., Fujihara S., Iwama H., Yamashita T., Kobayashi K., Kamada H., Morishita A., Kobara H., Mori H. (2015). Antitumor effect of metformin on cholangiocarcinoma: In vitro and in vivo studies. Oncol. Rep..

[55] Li Y., Zhang J., Ma H. (2014). Chronic inflammation and gallbladder cancer. Cancer Lett..

[56] Hirsch H.A., Iliopoulos D., Struhl K. (2013). Metformin inhibits the inflammatory response associated with cellular transformation and cancer stem cell growth. Proc. Natl. Acad. Sci. USA.

[57] Lee J.-W., Choi E.-A., Kim Y.-S., Kim Y., You H.-S., Han Y.-E., Kim H.-S., Bae Y.-J., Kim J., Kang H.-T. (2020). Metformin usage and the risk of colorectal cancer: A national cohort study. Int. J. Color. Dis..

[58] Kang J., Jeong S.-M., Shin D.W., Cho M., Cho J.H., Kim J. (2020). The Associations of Aspirin, Statins, and Metformin With Lung Cancer Risk and Related Mortality: A Time-Dependent Analysis of Population-Based Nationally Representative Data. J. Thorac. Oncol..

[59] Linkeviciute-Ulinskiene D., Patasius A., Kincius M., Zabuliene L., Smailyte G. (2020). Preexisting diabetes, metformin use and long-term survival in patients with prostate cancer. Scand. J. Urol..

[60] Wynn A., Vacheron A., Zuber J., Solomon S.S. (2019). Metformin Associated with Increased Survival in Type 2 Diabetes Patients with Pancreatic Cancer and Lymphoma. Am. J. Med. Sci..

[61] Wang Y., Maurer M.J., Larson M.C., Allmer C., Feldman A.L., Bennani N.N., Thompson C.A., Porrata L.F., Habermann T.M., Witzig T.E. (2019). Impact of metformin use on the outcomes of newly diagnosed diffuse large B-cell lymphoma and follicular lymphoma. Br. J. Haematol..

[62] Tseng C.-H. (2018). Metformin is associated with decreased skin cancer risk in Taiwanese patients with type 2 diabetes. J. Am. Acad. Dermatol..

[63] Cho Y.Y., Kang M.J., Kim S.K., Jung J.H., Hahm J.R., Kim T.H., Nam J.Y., Lee B.-W., Lee Y.-H., Chung J.H. (2018). Protective Effect of Metformin Against Thyroid Cancer Development: A Population-Based Study in Korea. Thyroid.

[64] Becker C., Jick S., Meier C.R., Bodmer M. (2017). Metformin and the risk of renal cell carcinoma: A case–control analysis. Eur. J. Cancer Prev..

[65] Kozak M.M., Anderson E.M., von Eyben R., Pai J.S., Poultsides G.A., Visser B.C., Norton J.A., Koong A.C., Chang D.T. (2016). Statin and Metformin Use Prolongs Survival in Patients With Resectable Pancreatic Cancer. Pancreas.

[66] Nevadunsky N.S., Van Arsdale A., Strickler H.D., Moadel A., Kaur G., Frimer M., Conroy E., Goldberg G.L., Einstein M.H. (2014). Metformin use and endometrial cancer survival. Gynecol. Oncol..

