# Supplementary material for: Effects of Metformin on Risk and Prognosis of Biliary Tract Cancer: A Systematic Review and Meta-Analysis
Source: Medicina (Kaunas). 2023 Feb 6;59(2):298. doi: 10.3390/medicina59020298 (PMC9967261; doi:10.3390/medicina59020298)
Supplement: Supplementary file 1 [file medicina-59-00298-s001.zip › medicina-2151071-supplementary.pdf]

Table S1. Search strategies for each database	
**PubMed**	((("Cholangiocarcinoma"[Mesh] OR Cholangiocarcinomas OR Cholangiocellular Carcinoma OR Carcinoma, Cholangiocellular OR Carcinomas, Cholangiocellular OR Cholangiocellular Carcinomas OR Extrahepatic Cholangiocarcinoma OR Cholangiocarcinoma, Extrahepatic OR Cholangiocarcinomas, Extrahepatic OR Extrahepatic Cholangiocarcinomas OR Intrahepatic Cholangiocarcinoma OR Cholangiocarcinoma, Intrahepatic OR Cholangiocarcinomas, Intrahepatic OR Intrahepatic Cholangiocarcinomas) OR (ampulla of Vater cancer OR Vater cancer OR Ampullary Carcinoma OR ampulla of Vater OR Ampulla Vater cancer) OR ("Biliary Tract Neoplasms"[Mesh] OR Biliary Tract Neoplasm OR Neoplasm, Biliary Tract OR Neoplasms, Biliary Tract OR Biliary Tract Cancer OR Biliary Tract Cancers OR Cancer, Biliary Tract OR Cancers, Biliary Tract OR Cancer of the Biliary Tract OR Cancer of Biliary Tract OR bile duct carcinoma OR biliary duct carcinoma) OR ("Gallbladder Neoplasms"[Mesh] OR Gallbladder Neoplasm OR Neoplasm, Gallbladder OR Neoplasms, Gallbladder OR Cancer of Gallbladder OR Gallbladder Cancers OR Gallbladder Cancer OR Cancer, Gallbladder OR Cancers, Gallbladder OR Gall Bladder Cancer OR Bladder Cancer, Gall OR Bladder Cancers, Gall OR Cancer, Gall Bladder OR Cancers, Gall Bladder OR Gall Bladder Cancers OR Cancer of the Gallbladder)) AND ("Metformin"[Mesh] or Dimethylbiguanidine or Dimethylguanylguanidine or Glucophage or Metformin Hydrochloride or Hydrochloride, Metformin or Metformin HCl or HCl, Metformin))
**Embase**	Query('biliary tract tumor'/exp OR 'biliary tract neoplasms':ab,ti OR 'biliary tract tumour':ab,ti OR 'neoplasm, biliary tract':ab,ti OR 'neoplasms, biliary tract':ab,ti OR 'biliary tract cancer':ab,ti OR 'biliary tract cancers':ab,ti OR 'cancer, biliary tract':ab,ti OR 'cancers, biliary tract':ab,ti OR 'cancer of the biliary tract':ab,ti OR 'cancer of biliary tractt':ab,ti OR 'bile duct carcinoma'/exp OR 'bile tract carcinoma':ab,ti OR 'biliary carcinoma':ab,ti OR 'biliary duct carcinoma':ab,ti OR 'biliary tract carcinoma':ab,ti OR 'carcinoma, intrahepatic bile duct':ab,ti OR 'cholangiocarcinoma':ab,ti OR 'cholangiocellular carcinoma':ab,ti OR 'cholangiolar carcinoma':ab,ti OR 'extrahepatic bile duct carcinoma':ab,ti OR 'extrahepatic biliary duct carcinoma':ab,ti OR 'extrahepatic cholangiocarcinoma':ab,ti OR 'gall duct carcinoma':ab,ti OR 'hilar cholangiocarcinoma':ab,ti OR 'intrahepatic bile duct carcinoma':ab,ti OR 'intrahepatic biliary duct carcinoma':ab,ti OR 'intrahepatic cholangiocarcinoma':ab,ti OR 'malignant cholangioma':ab,ti OR 'perihilar bile duct carcinoma':ab,ti OR 'perihilar biliary duct carcinoma':ab,ti OR 'perihilar cholangiocarcinomat':ab,ti OR 'vater papilla carcinoma'/exp OR 'ampulla of vater carcinoma':ab,ti OR 'ampulla vateri carcinoma':ab,ti OR 'ampullary carcinoma':ab,ti OR 'ampullovateric carcinoma':ab,ti OR 'cancer, papilla vateri':ab,ti OR 'carcinoma alcholedochoduodenal junction':ab,ti OR 'carcinoma, papilla vateri':ab,ti OR 'carcinoma, vater papilla':ab,ti OR

	'choledochoduodenal junction cancer':ab,ti OR 'papilla of vater carcinoma':ab,ti OR 'vater ampulla carcinomat':ab,ti OR 'gallbladder tumor'/exp OR 'gallbladder neoplasms':ab,ti OR 'gallbladder tumour':ab,ti OR 'tumor, gallbladder':ab,ti OR 'tumour, gallbladdert':ab,ti) AND ('metformin'/exp OR 'apophage':ab,ti OR 'aron':ab,ti OR 'benofomin':ab,ti OR 'dabex':ab,ti OR 'denkaform':ab,ti OR 'deson':ab,ti OR 'dextin':ab,ti OR 'diabetase':ab,ti OR 'diabetase s':ab,ti OR 'diabetformin':ab,ti OR 'diabetmin':ab,ti OR 'diabetmin retard':ab,ti OR 'diabetosan':ab,ti OR 'diabex':ab,ti OR 'diafat':ab,ti OR 'diaformin':ab,ti OR 'diaformina':ab,ti OR 'diaformina lp':ab,ti OR 'diametin':ab,ti OR 'diamin':ab,ti OR 'dianben':ab,ti OR 'diformin':ab,ti OR 'diformin retard':ab,ti OR 'dimefor':ab,ti OR 'dimethylbiguanide':ab,ti OR 'dimethyldiguanide':ab,ti OR 'dmgg':ab,ti OR 'dybis':ab,ti OR 'eraphage':ab,ti OR 'espa-formin':ab,ti OR 'euform retard':ab,ti OR 'fluamine':ab,ti OR 'flumamine':ab,ti OR 'fornidd':ab,ti OR 'fortamet':ab,ti OR 'glafornil':ab,ti OR 'glibudon':ab,ti OR 'glifage':ab,ti OR 'gliguanid':ab,ti OR 'glucaminol':ab,ti OR 'glucofage':ab,ti OR 'glucofago':ab,ti OR 'glucoform':ab,ti OR 'glucoformin':ab,ti OR 'glucohexal':ab,ti OR 'glucoless':ab,ti OR 'glucomet':ab,ti OR 'glucomin':ab,ti OR 'glucomine':ab,ti OR 'gluconil':ab,ti OR 'glucophage':ab,ti OR 'glucophage forte':ab,ti OR 'glucophage retard':ab,ti OR 'glucophage sr':ab,ti OR 'glucophage xr':ab,ti OR 'glucophage xr extended release':ab,ti OR 'glucophage-mite':ab,ti OR 'glucostop':ab,ti OR 'glucotika':ab,ti OR 'gludepatic':ab,ti OR 'glufor':ab,ti OR 'gluformin':ab,ti OR 'glukophage':ab,ti OR 'glumeformin':ab,ti OR 'glumet':ab,ti OR 'glumetza':ab,ti OR 'glupa':ab,ti OR 'glustress':ab,ti OR 'glyciphage':ab,ti OR 'glycomet':ab,ti OR 'glycon':ab,ti OR 'glycoran or glyformin':ab,ti OR 'glymet':ab,ti OR 'haurymellin':ab,ti OR 'hipoglucin':ab,ti OR 'i-max':ab,ti OR 'islotin':ab,ti OR 'jesacrin':ab,ti OR 'juformin':ab,ti OR 'la 6023':ab,ti OR 'la6023':ab,ti OR 'lyomet (drug)':ab,ti OR 'maformin':ab,ti OR 'meglucon':ab,ti OR 'meguan':ab,ti OR 'melbin':ab,ti OR 'melformin':ab,ti OR 'mellittin':ab,ti OR 'merckformin':ab,ti OR 'mescorit':ab,ti OR 'metaformin':ab,ti OR 'metfogamma':ab,ti OR 'metfoliquid geriasan':ab,ti OR 'metforal or metformax or metformin hydrochloride or metformina':ab,ti OR 'metformine':ab,ti OR 'metformine hcl':ab,ti OR 'methformin':ab,ti OR 'metiguanide':ab,ti OR 'metomin':ab,ti OR 'metphormin':ab,ti OR 'miformin':ab,ti OR 'n dimethylguanylguanide':ab,ti OR 'n dimethylguanylguanidine':ab,ti OR 'n, n dimethyl biguanidine':ab,ti OR 'n, n dimethylbiguanide':ab,ti OR 'n, n dimethylbiguanide retard':ab,ti OR 'n, n dimethylbiguanidine':ab,ti OR 'n, n dimethyldiguanide':ab,ti OR 'n, n dimethylguanylguanidine':ab,ti OR 'neoform':ab,ti OR 'nndg':ab,ti OR 'reglus-500':ab,ti OR 'riomet':ab,ti OR 'riomet er':ab,ti OR 'risidon':ab,ti OR 'siamformet':ab,ti OR 'siofor':ab,ti OR 'thiabet':ab,ti OR 'vimetrol':ab,ti OR 'walaphage':ab,ti)

**Web of science**	TS=(((Cholangiocarcinoma OR Cholangiocarcinomas OR Cholangiocellular Carcinoma OR Carcinoma, Cholangiocellular OR Carcinomas, Cholangiocellular OR Cholangiocellular Carcinomas OR Extrahepatic Cholangiocarcinoma OR Cholangiocarcinoma, Extrahepatic OR Cholangiocarcinomas, Extrahepatic OR Extrahepatic Cholangiocarcinomas OR Intrahepatic Cholangiocarcinoma OR Cholangiocarcinoma, Intrahepatic OR Cholangiocarcinomas, Intrahepatic OR Intrahepatic Cholangiocarcinomas) OR (ampulla of Vater cancer OR Vater cancer OR Ampullary Carcinoma OR ampulla of Vater OR Ampulla Vater cancer) OR (Biliary Tract Neoplasms OR Biliary Tract Neoplasm OR Neoplasm, Biliary Tract OR Neoplasms, Biliary Tract OR Biliary Tract Cancer OR Biliary Tract Cancers OR Cancer, Biliary Tract OR Cancers, Biliary Tract OR Cancer of the Biliary Tract OR Cancer of Biliary Tract OR bile duct carcinoma OR biliary duct carcinoma) OR (Gallbladder Neoplasms OR Gallbladder Neoplasm OR Neoplasm, Gallbladder OR Neoplasms, Gallbladder OR Cancer of Gallbladder OR Gallbladder Cancers OR Gallbladder Cancer OR Cancer, Gallbladder OR Cancers, Gallbladder OR Gall Bladder Cancer OR Bladder Cancer, Gall OR Bladder Cancers, Gall OR Cancer, Gall Bladder OR Cancers, Gall Bladder OR Gall Bladder Cancers OR Cancer of the Gallbladder)) AND (Metformin or Dimethylbiguanidine or Dimethylguanylguanidine or Glucophage or Metformin Hydrochloride or Hydrochloride, Metformin or Metformin HCl or HCl, Metformin))
**Cochrane library**	#1 MeSH descriptor: [Biliary Tract Neoplasms] explode all trees<br>#2 Neoplasm, Biliary Tract or Neoplasms, Biliary Tract or Biliary Tract Neoplasm or Cancer of Biliary Tract or Cancers, Biliary Tract or Cancer, Biliary Tract or Biliary Tract Cancers or Cancer of the Biliary Tract orBiliary Tract Cancer<br>#3 MeSH descriptor: [Cholangiocarcinoma] explode all trees<br>#4 Cholangiocarcinomas or Extrahepatic or Cholangiocarcinoma, Extrahepatic or Extrahepatic Cholangiocarcinomas or Extrahepatic Cholangiocarcinoma or Cholangiocellular Carcinoma or Carcinoma, Cholangiocellular or Carcinomas, Cholangiocellular or Cholangiocellular Carcinomas or Cholangiocarcinomas or Cholangiocarcinomas, Intrahepatic or Cholangiocarcinoma, Intrahepatic or Intrahepatic Cholangiocarcinoma or Intrahepatic Cholangiocarcinomas<br>#5 MeSH descriptor: [Gallbladder Neoplasms] explode all trees<br>#6 Cancer of the Gallbladder or Bladder Cancer, Gall or Cancer of Gallbladder or Gall Bladder Cancer or Gall Bladder Cancers or Bladder Cancers, Gall or Cancer, Gall Bladder or Cancers, Gall Bladder or Neoplasm, Gallbladder or Neoplasms, Gallbladder or Gallbladder Neoplasm or Gallbladder Cancer or Gallbladder Cancers or Cancer, Gallbladder or Cancers, Gallbladder<br>#7 Ampullary carcinoma

	#8 MeSH descriptor: [Metformin] explode all trees<br>#9 Dimethylbiguanidine or Dimethylguanylguanidine or Glucophage or Metformin HCl or Metformin Hydrochloride or HCl, Metformin or Hydrochloride, Metformin<br>#10 (#1 OR #2 OR #3 OR #4 OR #5 OR #6 OR #7) AND (#8 OR #9)

Table S2. Studies included in each meta-analysis

Meta-analysis group		Tseng et al. [1]	Wu et al. [2]	Yang et al. [3]	Gardini et al. [4]	Oh et al. [5]	Jong et al. [6]	Valent et al. [7]	Bonilla et al. [8]	McNamara et al. [9]	Sookaromdee et al. [10]	Chaiteerakij et al. [11]
**Risk of biliary tract cancer**		√				√	√	√	√		√	√
**Subgroups**	Diabetics	√				√	√	√				√
	General population										√	
	<65 years	√				√	√					√
	≥65 years											
	Cholangiocarcinoma										√	√
	Gallbladder cancer							√				
	Asia	√				√					√	
	Non-Asia						√	√				√
	N<100,000					√	√					√
	N≥100,000	√						√			√	
**Overall survival**		√	√	√	√					√		
**Subgroups**	Diabetics	√		√								
	General population				√					√		
	<65 years	√										
	≥65 years			√						√		
	Cholangiocarcinoma			√								
	Asia	√										
	non-Asia			√	√					√		
	<300			√	√							
	≥300	√			√					√		

**Disease-free survival**				√					√		

References:

1. Tseng, C.-H. Metformin and Biliary Tract Cancer in Patients With Type 2 Diabetes. *Front. Oncol.* **2020**, *10*, 587666. https://doi.org/10.3389/fonc.2020.587666.
2. Wu, J.; Zhou, Y.; Wang, G. Metformin Use and Survival in Patients with Advanced Extrahepatic Cholangiocarcinoma: A Single-Center Cohort Study in Fuyang, China. *Gastroenterol. Res. Pract.* **2021**, *2021*, 1–8. https://doi.org/10.1155/2021/9468227.
3. Yang, Z.; Zhang, X.; Roberts, R.O.; Roberts, L.R.; Chaiteerakij, R. Metformin does not improve survival of cholangiocarcinoma patients with diabetes. *Hepatology* **2016**, *63*, 667–668. https://doi.org/10.1002/hep.27821.
4. Casadei-Gardini, A.; Filippi, R.; Rimini, M.; Rapposelli, I.G.; Fornaro, L.; Silvestris, N.; Aldrighetti, L.; Aimar, G.; Rovesti, G.; Bartolini, G.; et al. Effects of Metformin and Vitamin D on Clinical Outcome in Cholangiocarcinoma Patients. *Oncology* **2021**, *99*, 292–299. https://doi.org/10.1159/000512796.
5. Oh, T.K.; Song, I.-A. Metformin Use and the Risk of Cancer in Patients with Diabetes: A Nationwide Sample Cohort Study. *Cancer Prev. Res.* **2020**, *13*, 195–202. https://doi.org/10.1158/1940-6207.capr-19-0427.
6. de Jong, R.G.; Burden, A.M.; de Kort, S.; van Herk-Sukel, M.P.; Vissers, P.A.; Janssen, P.K.; Haak, H.R.; Masclee, A.A.; de Vries, F.; Janssen-Heijnen, M.L. No Decreased Risk of Gastrointestinal Cancers in Users of Metformin in The Netherlands; A Time-Varying Analysis of Metformin Exposure. *Cancer Prev. Res.* **2017**, *10*, 290–297. https://doi.org/10.1158/1940-6207.capr-16-0277.
7. Valent, F. Diabetes mellitus and cancer of the digestive organs: An Italian population-based cohort study. *J. Diabetes its Complicat.* **2015**, *29*, 1056–1061. https://doi.org/10.1016/j.jdiacomp.2015.07.017.
8. Bonilla, L.M.; Schleck, C.; Harmsen, W.; Therneau, T.; Sadr-Azodi, O.; Roberts, L.R.; Brusselaers, N. 3437 Associations of aspirin, non-aspirin NSAIDs, statins, and metformin with risk of biliary cancer: A Swedish population-based cohort study. *J. Clin. Transl. Sci.* **2019**, *3*, 35–35. https://doi.org/10.1017/cts.2019.85.
9. McNamara, M.G.; Aneja, P.; Le, L.W.; Horgan, A.M.; McKeever, E.; Knox, J.J. Effects of statin, aspirin, or metformin use on recurrence free and overall survival in patients with biliary tract cancer (BTC). *J. Clin. Oncol.* **2014**, *32*, 303–303. https://doi.org/10.1200/jco.2014.32.3_suppl.303.
10. Sookaromdee, P.; Wiwanitkit, V. Decreased risk of cholangiocarcinoma in diabetic patients treated with metformin. *J. Cancer Res. Ther.* **2020**, *16*, 82–S83. https://doi.org/10.4103/jcrt.jcrt_368_18.
11. Chaiteerakij, R.; Yang, J.D.; Harmsen, W.S.; Slettedahl, S.; Mettler, T.A.; Fredericksen, Z.S.; Kim, W.R.; Gores, G.J.; Roberts, R.O.; Olson, J.E.; et al. Risk factors for intrahepatic cholangiocarcinoma: Association between metformin use and reduced cancer risk. *Hepatology* **2012**, *57*, 648–655. https://doi.org/10.1002/hep.26092.
